# The Effect of Ligature Type on Lateral Tooth Movement during Orthodontic Treatment with Lingual Appliances—An In Vitro Study

**DOI:** 10.3390/ma15093365

**Published:** 2022-05-07

**Authors:** Elisabeth Reichardt, Steffen Decker, Michel Dalstra, Prasad Nalabothu, Markus Steineck, Leandro Fernandez, Carlalberta Verna

**Affiliations:** 1 Department of Pediatric Oral Health and Orthodontics, University Center for Dental Medicine UZB, University of Basel, Mattenstrasse 40, 4058 Basel, Switzerland; decker.steffen.1982@gmail.com (S.D.); michel.dalstra@dent.au.dk (M.D.); prasad.nalabothu@unibas.ch (P.N.); markus.steineck@uzb.ch (M.S.); leandrof@telefonica.net (L.F.); carlalberta.verna@uzb.ch (C.V.); 2 Department of Odontology and Dental Health, Section of Orthodontics, University of Aarhus, Vennelyst Blvd. 9, 8000 Aarhus, Denmark

**Keywords:** lingual orthodontic appliances, orthodontic treatment, tip control, ligation

## Abstract

(1) Background: One of the most challenging parts in lingual orthodontics is the control and correction of the tip of anterior teeth, due to the occlusal open vertical slot of the incisors in lingual systems. The presented experimental in-vitro study was performed to determine the maximal tipping moment of the anterior teeth between two types of lingual brackets, the Incognito™ Appliance System (Incognito, TOP-Service, Bad Essen, Germany) and Tip-Bar™ system (Incognito, TOP-Service, Bad Essen, Germany). Furthermore, twelve different ligation methods and two different ligature materials were investigated. (2) Methods: The measurement was performed by assessing the stiffness and ultimate strength of the ligature in a uniaxial material testing machine (Instron, Norwood, MA, USA) using a 0.025 × 0.018 inch stainless steel wire. (3) The results showed that the highest precision for control tipping of anterior teeth was determined for the 0.010 inch Stainless Steel Tie (Pelz and Partner). Furthermore, the Tip-Bar™ brackets increased the maximal moment by 33.8% for elastic and steel ligatures. (4) Conclusions: The lateral tooth movement is highly dependent on the type of ligature and applied material during orthodontic treatment with lingual appliances. The use of 0.010 inch steel ligatures and the Tip-Bar™ bracket design results in better alignment in the anterior teeth segment.

## 1. Introduction

The lingual orthodontic bracket systems have received rising popularity and shall serve as an option that does not affect the aesthetics during orthodontic treatment [1]. The Incognito™ Appliance System (IAS, Incognito, TOP-Service, Bad Essen, Germany) was designed to make orthodontic treatment more predictable and efficient, as each bracket pad and body conforms to the patient’s dental anatomy, achieving the lowest bracket profile for optimal patient comfort. At the moment, the Incognito™ Appliance System (IAS) implements fully digital workflow and production process, starting with an intraoral scan or standard PVS impression, virtual treatment planning with the aid of Treatment Management Portal (TMP), digital lab process, and, finally, the bonding of the appliance with a digitally made clear precision tray (CPT) [2]. The IAS has a slot of the highest precision, in comparison to diverse alternative lingual brackets [3]. The precision of the slot, combined with a full thickness ribbonwise wire and lingual ligatures, provides precise control of the tooth position on three planes. A ribbonwise-shaped, rectangular orthodontic arch wire is applied to the dental arches, so that its widest dimension is parallel to the labial or buccal surfaces of the teeth. For this reason, the wire dimension in the IAS system is considered to be 0.025 × 0.018 inch [2].

One of the most challenging parts in lingual orthodontics is the control and correction of the tip in anterior teeth, due to the occlusal open vertical slot of anterior teeth, type of ligation, material, and wire, which has to be fully seated in the slot [4]. The term crown tip describes the crown angulation—the angle formed by the facial axis of the clinical crown (FACC) and a line perpendicular to the occlusal plane. A tooth can have a positive or negative crown angulation (tip). The ideal crown angulation or tip is defined by Andrews’s six keys to normal occlusion [5]. Tipping is a tooth movement, which alters the angulation of the long axis of the tooth, either in the sagittal or horizontal planes. The center of resistance (Cres) of an anterior front tooth is at 40 percent of the root length [6]. Therefore, when applying a perpendicular force at the bracket, the crown will tip in the direction of the force, depending on the force vector [4].

In orthodontic treatment, tipping can be present due to earlier natural tooth movements. It can also be created owing to loss of control during orthodontic tooth movements by disengaging of the wire. Furthermore, the force can be applied away from the Cres, or the stiffness of the wire may not be sufficient [4].

**Figure 1:**
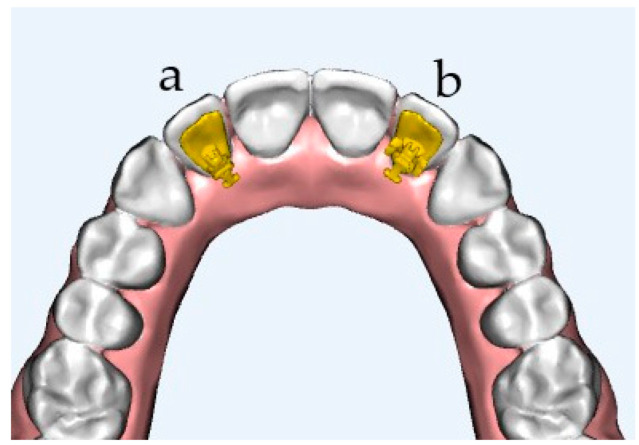
Bracket design (**a**): Incognito™ Appliance System (IAS) and (**b**): Tip-Bar™ System (Incognito, TOP-Service, Bad Essen, Germany).

Controlling and correcting the tip requires an arch wire, fully seated into the vertical slot. The best control of the tip will be achieved by a greater wideness of the slot and higher moment of force [7]. Hence, the change in the tip requires a stronger ligation, which can be created by a special ligation, called power tie, in order to fully seat the wire in the vertical slot. Moreover, the company 3M Unitek developed a new Tip-Bar™ bracket design, with extensions mesially and distally of the slot base to increase the slot width and, hence, the moment compared to a standard Incognito™ bracket (Figure 1). Due to these extended bracket slots, 3M Oral Care claims to achieve 75 percent larger tip moment.

**Figure 2:**
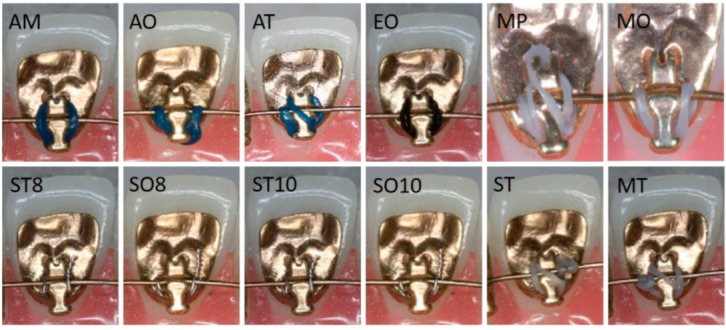
Overview of the different types of ligatures. AM: Alastik module, AO: Alastik over tie, AT: Alastik tipping tie, EO: EasyOn, MT: modified tipping tie, MO: Morita over tie, MP: Morita power tie, ST8: steel tie (0.008 inch), ST10: steel tie (0.010 inch), SO8: steel over tie (0.008 inch), SO10: steel over tie (0.010 inch), ST: Sugiyama tipping tie.

The aim of the current experimental in vitro study was to determine the maximal tipping moment of the anterior teeth among two types of lingual brackets: the IAS (Incognito, TOP-Service, Bad Essen, Germany) and Tip-Bar™ system (Incognito, TOP-Service, Bad Essen, Germany). Additionally, twelve different ligation methods and two different ligature materials were investigated (Figure 2, Table 1). It was hypothesized that the bracket morphology and ligature type have an influence on lateral tooth movement during orthodontic treatment with lingual appliances.

## 2. Materials and Methods

**Figure 3:**
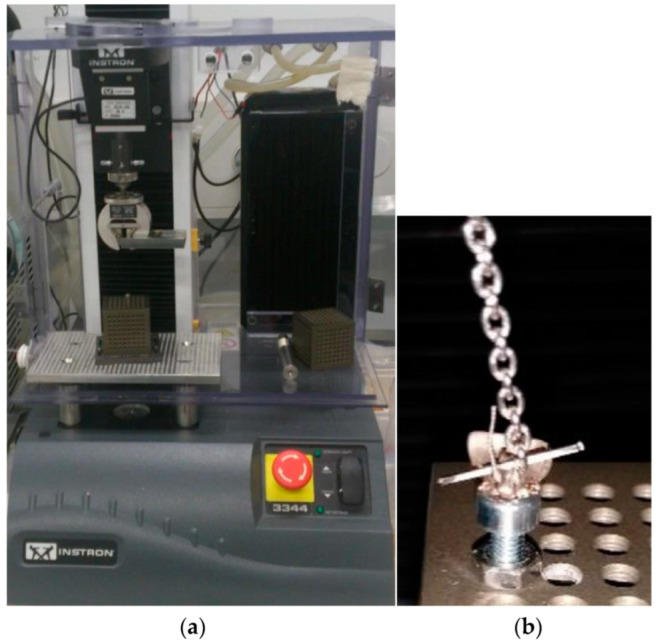
Experimental set-up. (**a**) Uniaxial material testing machine (Instron, Norwood, MA, USA). (**b**) Measurement of stiffness and strength after fixing the screw with bracket and wire to the testing machine. The wire was taken out of the slot using a chain.

**Figure 4:**
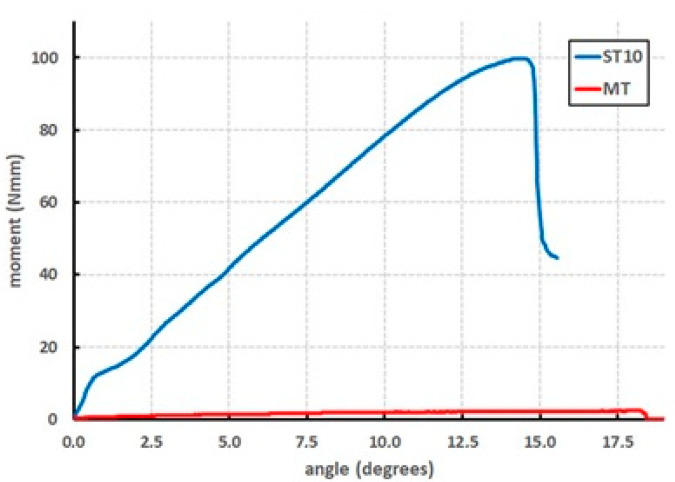
Two examples of moment/angle test curves. Blue: steel tie (0.010 inch) (ST10), featuring among the highest stiffness and maximal moment values. Red: modified tipping tie (MT), featuring among the lowest stiffness and maximal moment values.

The experimental setup was as follows: two different bracket designs were examined: the Incognito™ Appliance System (Incognito, TOP-Service, Bad Essen, Germany) and Tip-Bar™ system (Incognito, TOP-Service, Bad Essen, Germany). The orthodontic lingual bracket has a base that is connected to the tooth and defined slot that is made for the orthodontic arch wire (Figure 1). The new developed Tip-Bar™ Bracket has additional two tip bars beside the bracket slot on both sides (Figure 1). The Incognito™ bracket has a slot width of 2.5 mm, and the Tip-Bar™ has a slot width of 4.1 mm; both brackets have a slot size of 0.018 inch. A slot-filling 0.025 × 0.018 inch stainless steel wire was inserted in the slot, and the investigation was carried out via the insertion of twelve different ligatures (Figure 2, Table 1). The ligatures were substituted after each measurement, and the test of ligature type resp. bracket combination was repeated ten times, always by the same operator. The control of tip of the anterior teeth was measured by determining the stiffness and ultimate strength of the ligature in the uniaxial material testing machine (Instron, Norwood, MA, USA) (Figure 3). For this purpose, a single anterior central incisor bracket of each bracket system was soldered onto a mounting screw. After fixing the screw with bracket and wire to the testing machine, the wire was taken out of the slot using a steel chain (Figure 3), and the resulting force-displacement data were recorded. Next, the data were transformed into moment-angulation data and subsequently plotted in curves (Figure 4). The slopes of these curves represent the stiffness of the ligature, while the top point is a measure of the ultimate strength, and these variables were used for further analysis. 

### Statistics

Descriptive statistic was used to calculate the means and standard deviations of the stiffness and strength for the various ligature type and bracket combination. The normality of the data was investigated using QQ plots. One-way ANOVA was used to assess any statistically significant differences between the 12 ligature types for the two bracket systems. The minimal required sample size of seven was estimated using a power test for one-sided ANOVA with: number of groups = 24, effect size = 0.4, significance level = 0.05, and power = 0.8. The Student–Newman–Keuls post hoc test was used to identify significant differences between the different ligature types (subsets) for either the IAS or the Tip-Bar™ brackets. All statistical analyses were performed using SPSS (Version 24, IBM, Armonk, NY, USA).

## 3. Results

The results of the present study showed a large range in stiffness and strength values for the various ligature types (Table 2 and Table 3). Statistically significant differences in the materials were found between the standard brackets IAS (Incognito, TOP-Service, Bad Essen, Germany) and Tip-Bar™ (Incognito, TOP-Service, Bad Essen, Germany). The stiffness and strength values were smaller for the Incognito™ brackets, as compared to the Tip-Bar™ brackets. Furthermore, the results showed that the Tip-Bar™ brackets increased the maximum moment by 33.8%, using elastic and steel ligatures.

### 3.1. Stiffness (Nmm/Degree)

The stiffness of the IAS system ranged from 0.3 ± 0.1 Nmm/degree (mean ± SD) for MT ligatures to 6.6 ± 0.9 Nmm/degree for ST10 ligatures (Table 2). The stiffness of Tip-Bar™ ligatures ranged from 0.98 ± 0.21 Nmm/degree for MO to 9.55 ± 0.81 Nmm/degree for ST10 ligatures (Table 2). The results showed significantly higher stiffness values for the Tip-Bar™ brackets, compared to the standard IAS bracket, for all ties, except the AO (*p* < 0.05). 

### 3.2. Strength (Maximal Moment, Nmm)

The strength of the IAS system ranged from 5.2 ± 1.82 Nmm for MT ligatures to 109.17 ± 11.13 Nmm for ST10 ligatures (Table 3). The maximal moment for the ligature at Tip-Bar™ ranged from 12.67 ± 2.7 Nmm for MO to 148.62 ± 20.14 Nmm for ST10 ligatures (Table 3). The highest precision for control of tipping of the anterior teeth was ascertained for the ST10. The results showed significantly higher maximal moments for the Tip-Bar™ brackets, compared to the standard IAS brackets, for all ties (*p* < 0.05).

### 3.3. Differences between Ligature Types

The stiffness and strength values for the steel ligatures were always significantly larger, compared to the elastomer ligatures. 

#### 3.3.1. Elastomer Ligatures

The AT ligatures showed higher stiffness and strength values than the other elastomer ligature types. Additionally, significant differences were found for the Tip-Bar™ brackets. In regard to the analysis of the maximal moment, a similar result was observed for the AT ligatures, which demonstrated higher maximal moments, due to the Tip-Bar™ group.

#### 3.3.2. Stainless Steel Ligatures

The results of the investigated stainless steel ligatures showed significantly higher stiffness and maximal moment values for ST10 ligatures, compared to the other steel ligatures, for both the IAS and Tip-Bar™ groups.

## 4. Discussion

The development of fully customized lingual systems using CAD-CAM technology has reduced limitations and allowed clinicians to successfully treat orthodontic cases [8]. Many of the problems with conventional lingual bracket systems have been overcome with the recently developed Incognito™ lingual bracket design [9]. Moreover, the result of the orthodontic treatment is significantly influenced by the use of suitable materials, and the implementation is particularly important in the esthetically visible area of the anterior teeth [10]. Sufficient ligation force is necessary to seat the wire into the base of the slot, especially where visible tip change is required [11]. The present study investigated the mechanical properties of various ligature methods, with two different bracket combination. Therefore, the measurement through the stiffness, as a quantification of the generated moment by an increase in tipping angle of one degree, was examined. Additionally, the strength, as a quantification of the maximal moment before rupture of the ligature, was performed. The results showed that, in general, steel ligatures presented higher values of stiffness and strength than elastomeric ligatures.

### 4.1. Steel Ligatures

There have been continuous attempts to improve smile esthetic by controlling lateral tooth movement [12,13]. The present study showed that the steel ligatures are the most stable and secure ligatures for adequate tip in the anterior teeth. The highest precision for tipping control of anterior teeth, due to stiffness, was determined for the ST10, followed by SO10. These findings agree with the results of a study from Romano, where the maximum engagement of the wire was also achieved by steel ligatures [14]. Moreover, the results of the maximal moment (Nmm) showed a statistically significant increase for ST10 and SO10 within the Tip-Bar™ group, compared to the IAS group (Table 2 and Table 3). Hence, the use of 0.010 inch steel ligatures and the Tip-Bar™ bracket improve the orthodontic outcome in the esthetically visible area by enhanced tip control.

### 4.2. Elastics

Elastomeric ligatures are easier and faster to insert and, therefore, usually preferred during lingual orthodontic treatment. Therefore, one advantage of using elastic ligatures is chair-time reduction for the practitioner and practice [15]. Elastic double over ties were routinely used for ligation of the anterior brackets. When a change in tip is required, the power tie (Figure 2, Morita Powerchain) is still recommended as the most effective method for achieving full seating of the wire in the vertical slot [2]. Although effective, these types of ligatures are difficult to insert, and they induce high friction [2].

Among the elastomer ligatures, the AT ligatures showed significant higher stiffness and strength values, as compared to the other ligature types. To correctly perform a tip, the results showed that an AT ligature inserted the wire more exactly than other elastic ligatures (Table 2 and Table 3). Moreover, AT ligatures significantly improved lateral tooth movement when the Tip-Nar™ brackets were used, as compared to standard brackets (Table 2 and Table 3). Interestingly, Hirose presented a so-called “tip-chain”, in order to prevent the anterior teeth from tipping in extraction cases, which could also be performed in anterior space closure cases to change the tip of individual teeth [4]. The “tip-chain” ligature was not included in this study, since the study set up was built to analyze single lingual brackets. 

Therefore, all investigated ligatures, apart from the AM and the EO power rings, are quite challenging to place, and the success is dependent on the skill of the orthodontist. This has a substantial influence on the clinical outcome, accuracy, and completion of orthodontic treatment [2]. In addition, the engagement of the wire, use of different ligatures, and design of the bracket is also important for enhancing better tip control in the anterior teeth during lingual therapy. Hence, the results showed that the Tip-Bar™ brackets increased the maximum moment by 33.8% using elastic and steel ligatures. The authors concluded that the use of this bracket design leads to a better alignment in the anterior segment and sufficient control of tip during lingual treatment.

### 4.3. Resistance to Sliding

Resistance to sliding is a critical factor in fixed orthodontic appliance therapy, especially for the treatment of closing gaps after tooth extraction [11]. Binding forces increase rapidly once the critical contact angle is exceeded [16]. Tipping of the lateral incisors can occur in non-extraction and, especially, in extraction cases. Inami and colleagues recognized, in their case report, a tipping of the anterior laterals after closing four premolar extraction gaps with the Incognito™ lingual system. They indicated that vertical slots in the anterior region of the appliance can control rotation and labiolingual tipping during retraction, while mesiodistal tipping readily occurs if the tie is inadequate [9]. The amount of tipping and rotation depends on the difference between the sizes of the arch wire and the bracket slot. The use of smaller arch wires generates less resistance to sliding in lingual orthodontics and, consequently, less tipping control. However, the engagement of the wire in the presence of a small interbracket distance, especially in the anterior area, creates a greater angle and leads to an increasing resistance to sliding and higher retraction force [17]. 

Ligation of the arch wire has a critical influence on the friction values [17]. In the study of Romano and colleagues, a double over tie were described as the most common form of ligation with the Ormco-Kurz seventh generation brackets [14]. This type of ligation generates high friction, especially in lingual orthodontics, where the saliva is in direct contact with the brackets and elastics [18]. In the study of Pereira and colleagues, a stainless steel arch wire static friction in active and passive self-ligating lingual, and conventional brackets with second order angulations were tested. The results showed that elastic modules lose force faster in an oral environment to correct the position of the teeth than steel ligatures [19]. Supporting these findings, our results showed that the stainless steel ligature ST10 delivered the highest force and showed the greatest stability, in order to fully seat the wire in the vertical slot, however, without the presence of saliva. Leander and colleagues described that friction can be reduced by super slick ligatures because of their hydroxyapatite coating (Metafix), which minimize friction, adhesion of residues, and plaque [20]. However, this was not found in the study of Pereira and colleagues, where ligatures did not cause less friction, supporting the results of other studies, which evaluated different kinds of elastic ligatures [19]. Furthermore, some studies showed greater friction than those of self-ligating brackets and metal ligatures [17,21,22].

### 4.4. Arch Wire Dimension and Limitations of the Study

The most commonly used wire for torque control in lingual orthodontics is a 0.017 × 0.025 inch TMA wire in the 0.018 inch slot, with a torque loss of approximately 6 degrees [23]. Filling the bracket slot by incrementally increasing the wire cross section has been the basic mechano-therapeutic sequence for many fixed appliance protocols [24]. However, under clinical conditions, the cross section of the final arch wire does not reach the full size of the slot. A recently published survey reported that technical difficulties are one of the most common reasons for not using lingual appliances in practicing orthodontists in the United States [25]. Experience and sufficient treatment time are, therefore, an important factor for the successful insertion of the final arch wire [25]. Hence, the present study is limited by the investigation of a full-sized stainless-steel wire (0.025 × 0.018 inch) and examination of a single bracket set up. The investigation did not reflect a general clinical situation, because of the difficulty in inserting large rectangular wires into the bracket slot by orthodontists [14]. However, since the aim of the study was to compare the bracket design and ligature types for tip control, the choice of the ligature and bracket morphology will not change with the use of smaller wire sizes. Moreover, single point contacts also exist on each ligature and bracket combination when, clinically, more teeth are included in orthodontic treatment.

In summary, the authors conclude that the use of 0.010 inch steel ligatures and the Tip-Bar™ bracket design improve the orthodontic outcome by tip control and can be recommended for clinical application.

## 5. Conclusions

The use of lingual orthodontics has increased, due to the development of more advanced technologies. The result of orthodontic treatment is significantly influenced by the bracket design and precision of the ligatures, which improve the orthodontic outcome in esthetically visible areas. The present study showed that the 0.010 inch steel tie (ST10) ligature is the most stable and secure ligature available in the lingual system for adequate tip control in anterior teeth. Among elastic ligatures, the AT ligature showed the most precise engagement of the stainless steel wire (0.025 × 0.018 inch), compared to other ligatures. Furthermore, the Tip-Bar™ brackets increased the maximum moment by 33.8%. The use of 0.010 inch steel ligatures and the Tip-Bar™ bracket design results in better alignment in the anterior teeth segment.

## Figures and Tables

**Table 1:** Overview of the employed different ligation types.

Identifier	Name	Abbreviation	Producer
1	Alastik module	AM	3M Oral Care
2	Alastik over tie	AO	3M Oral Care
3	Alastik tipping tie	AT	3M Oral Care
4	EasyOn	EO	Pelz and Partner
5	Modified tipping tie	MT	Rocky Mountain Orthodontics
6	Morita over tie	MO	Rocky Mountain Orthodontics
7	Morita power tie	MP	Rocky Mountain Orthodontics
8	Steel tie (0.008 inch)	ST8	Pelz and Partner
9	Steel tie (0.010 inch)	ST10	Pelz and Partner
10	Steel over tie (0.008 inch)	SO8	Pelz and Partner
11	Steel over tie (0.010 inch)	SO10	Pelz and Partner
12	Sugiyama tipping tie	ST	Rocky Mountain Orthodontics

**Table 2:** Overview of the stiffness (mean and SD) of the various ligature types and bracket combinations. The *p* values (*) refer to a comparison between the IAS and Tip-Bar™ brackets.

Stiffness (Nmm/Degree)					
Ligature	IAS		Tip-Bar™		
	Mean	SD	Mean	SD	*p*
AM	0.78	0.06	1.12	0.07	0.0000 *
AO	1.09	0.27	1.26	0.19	0.1054 *
AT	1.24	0.27	2.60	0.35	0.0000 *
EO	0.77	0.10	1.11	0.06	0.0000 *
MP	1.01	0.32	1.62	0.16	0.0000 *
MO	0.76	0.16	0.98	0.21	0.0149 *
ST8	6.06	0.78	7.14	0.54	0.0021 *
SO8	6.01	0.69	7.42	1.03	0.0020 *
ST10	6.61	0.93	9.55	0.81	0.0000 *
SO10	5.67	1.08	9.58	0.76	0.0000 *
ST	0.41	0.13	1.09	0.57	0.0018 *
MT	0.30	0.11	1.18	0.26	0.0000 *

**Table 3:** Overview of the maximal moment (mean and SD) of the various ligature types and bracket combinations. The *p* values (*) refer to a comparison between the IAS and Tip-Bar™ brackets.

Maximal Moment (Nmm)					
Ligature	IAS		Tip-Bar™		
	Mean	SD	Mean	SD	*p*
AM	11.55	0.91	15.38	1.59	0.0000 *
AO	13.82	1.27	17.02	2.65	0.0029 *
AT	17.55	3.82	36.87	5.81	0.0000 *
EO	11.53	1.42	15.80	1.18	0.0000 *
MP	14.20	4.98	22.75	2.62	0.0001 *
MO	9.20	2.13	12.67	2.70	0.0051 *
ST8	90.26	9.25	107.37	9.52	0.0007 *
SO8	85.58	9.71	111.14	17.54	0.0008 *
ST10	109.17	11.13	148.62	20.14	0.0000 *
SO10	85.02	21.23	136.32	32.85	0.0006 *
ST	6.87	2.05	15.35	8.45	0.0064 *
MT	5.20	1.81	12.82	3.49	0.0000 *
